# Frequency of CYP2B6 Alleles in Major Iranian Ethnicities, Affecting Response to Efavirenz

**DOI:** 10.1155/2022/5754776

**Published:** 2022-10-19

**Authors:** Parham Mardi, Bahareh Tavakoli-Far, Samira Sheibani Nia, Roshanak Jazayeri, Massoud Houshmand

**Affiliations:** ^1^Student Research Committee, Alborz University of Medical Sciences, Karaj, Iran; ^2^Dietary Supplements and Probiotic Research Center, Alborz University of Medical Sciences, Karaj, Iran; ^3^Department of Physiology and Pharmacology, School of Medicine, Alborz University of Medical Sciences, Karaj, Iran; ^4^Department of Medical Genetics, National Institute of Genetic Engineering and Biotechnology, Tehran, Iran; ^5^Non-communicable Diseases Research Center, Alborz University of Medical Sciences, Karaj, Iran

## Abstract

**Introduction:**

Efavirenz is an antihuman immunodeficiency virus (HIV) drug metabolized by cytochrome P450 2B6 (CYP2B6) enzyme. Cytochrome P450 2B6 is an enzyme that in humans is encoded by the CYP2B6 gene. Polymorphisms of this gene play a crucial role in the metabolism of drugs such as Efavirenz. This study aims to evaluate the frequency of three clinically significant CYP2B6 polymorphisms (CYP2B6^*∗*^6 (516G > T), CYP2B6^*∗*^4 (785A > G), and CYP2B6^*∗*^5 (1459C > T)) in three major Iranian ethnicities.

**Methods:**

One hundred forty-seven participants from three main Iranian ethnicities were included in this study. After DNA extraction, CYP2B6^*∗*^6 (516G > T), CYP2B6^*∗*^4 (785A > G), and CYP2B6^*∗*^5 (1459C > T) were genotyped using tetra-primer amplification refractory mutation system polymerase chain reaction (ARMS-PCR).

**Results:**

The frequency of the mutated allele in the Iranian population for CYP2B6^*∗*^6 (516G > T) was 41.50 (95% CI: 35.81, 47.36), which was significantly lower than in Kurds (59.62, 95% CI: 45.10, 72.99). Similarly, Kurds had a higher frequency of mutated allele of CYP2B6^*∗*^5 (1459C > T) (46.15%, 95% CI: 32.23, 60.53) than in Iranians (24.49%, 95% CI: 19.68, 29.82). The frequency of A and G alleles of CYP2B6^*∗*^4 (785A > G) was 62.59% (95% CI: 56.78, 68.13) and 37.41 (95% CI: 31.87, 43.22), respectively.

**Conclusion:**

Kurds are at higher risk of adverse drug reactions (ADRs) and insufficient anti-HIV response compared to other Iranians.

## 1. Introduction

Penalized medicine is an approach that evaluates and manages patients based on their predicted response to treatment. This approach is also beneficial to predict and prevent therapeutic resistance, which can enhance patients' outcomes. One of the main components of this approach is to predict the drugs' metabolism pace and consequently drug response and toxicity [1, 2]. Different pathways have been identified for the metabolism of drugs. One of the crucial pathways is cytochrome P450, which comprises different enzymes [3, 4]. One of the major components of cytochrome P450 is CYP2B6, which is responsible for the metabolism of drugs such as antiretrovirals such as efavirenz [5, 6].

Efavirenz ((S)-6-chloro-4-(cyclopropylethynyl)-1,4-dihydro-4-(trifluoromethyl)-2H-3,1-benzoxazin-2-one) is one of the nonnucleoside reverse transcriptase inhibitors, which is prescribed as first-line therapy in patients diagnosed with human immunodeficiency virus (HIV) infection. Antiretroviral medications have transformed HIV infection into a chronic disease, which can be managed effectively [7]. Efavirenz, which is a noncompetitive inhibitor of HIV-1 reverse transcriptase, is one of the most effective drugs prescribed for HIV infection [8].

Efavirenz is highly potent; nevertheless, it is attributed to several adverse effects including, QT interval (the time from the start of the *Q* wave to the end of the *T* wave in electrocardiogram) prolongation, dyslipidemia, hepatotoxicity, and neuropsychiatric side effects [8]. Although the drug is no longer recommended as the preferred initial antiretroviral therapy (ART) regimen for nonpregnant adults due to its neuropsychiatric adverse effects, it is still recommended in selected patients based on the New York State Department of Health (NYSDOH) acquired immunodeficiency syndrome (AIDS) institute guideline (AI) [9]. Furthermore, unlike in the United States, efavirenz is a vital part of the first-line therapeutic guideline in middle and low-income countries such as Iran and it is prescribed in almost all patients diagnosed with HIV infection [10]. Side effects of efavirenz are one of the biggest challenges for health services in managing HIV-positive patients [11]. Evaluation of the pharmacokinetics of efavirenz is the key in managing its adverse drug reactions (ADRs) [12].

**Figure 1:**
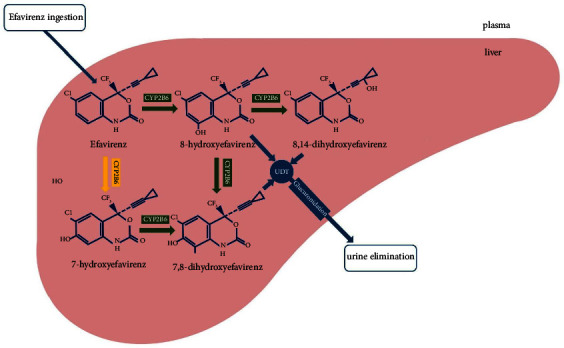
Schematic representation of efavirenz metabolism in the liver. It should be noted that the main route of efavirenz metabolism is 8-hydroxyefavirenz, which is predominately formed by CYP2B6. UGT, uridine 5′-diphospho-glucuronosyltransferase.

This drug initially transforms to its primary metabolite, 8-hydroxyefavirenz, in the liver mainly by cytochrome P450 2B6 (CYP2B6) enzyme, followed by the formation of 8,14-dihydroxyefavirenz chiefly by the same drug-metabolizing P450 2B6 [13].  Figure 1 concisely shows the metabolic pathway of efavirenz pharmacokinetics [13]. CYP2B6 is encoded by the CYP2B6 gene, and its various polymorphisms, including CYP2B6^*∗*^6 (516G > T), CYP2B6^*∗*^4 (785A > G), and CYP2B6^*∗*^5 (1459C > T), are clinically relevant for HIV-infected patients treated with efavirenz. For instance, the TT genotype for CYP2B6^*∗*^6 (516G > T) is associated with increased EFV plasma concentrations, reduced clearance, and consequently increased efavirnez exposure compared to the GG or GT genotype [14, 15]. In other words, patients' genotype for some of the CYP2B6 polymorphisms predicts the pace of drug metabolism in their bodies.

A recent study indicated that CYP2B6 polymorphisms are ethnically and geographically diverse among different populations, which results in differences in drugs, especially antiretrovirals, pharmacokinetics, and therapy outcomes [16]. Iran is a geographically diverse, and multiethnic country; Fars, Turk (Azerbaijanis), and Kurds comprise about 80 percent of the country's population [17]. The genotype and allele frequency of CYP2B6 polymorphisms have not been studied in any of its major ethnicities. This study aims to evaluate the frequency of three clinically important CYP2B6 polymorphisms (CYP2B6^*∗*^6 (516G > T), CYP2B6^*∗*^4 (785A > G), and CYP2B6^*∗*^5 (1459C > T)) in three major Iranian ethnicities.

## 2. Methods

### 2.1. Ethical Compliance

All patients consented to participate in genetic and molecular analyses and consented to publish the results. This study was verified by the Alborz University of Medical Sciences Ethical Committee (IR.ABZUMS.REC.1398.121).

### 2.2. Sample Collection and DNA Extraction

One hundred and forty seven participants were included in this study, comprising 26 Kurd, 52 Turk, and 69 Fars participants. Included participants aged between 18 and 77 years (median = 43, interquartile range (IQR) = 30 to 55). It should be noted that the number of participants from each ethnic group was calculated based on the ethnic composition of Iran [18]. Two milliliters of venous blood were gathered from each participant and drawn into a tube that contains ethylenediaminetetraacetic acid as an anticoagulant followed by DNA extraction using a molecular biological system transfer (MBST) salting-out kit (CinnaGen, Tehran, Iran) from the blood. Extracted DNA was stored at −20°C before genotyping.

### 2.3. Genotyping

**Figure 2:**
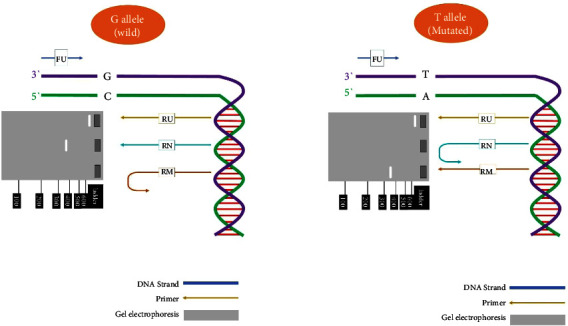
Process of SNP genotyping using Tetra-ARMS-PCR for CYP2B6^*∗*^6 (516G > T) in not-mutated (a) and mutated (b) DNAs. PCR product lengths of CYP2B6^*∗*^6 (516G > T) were FU + RU = 600, FU + RN = 353, and FU + RM = 353.

CYP2B6^*∗*^6 (516G > T), CYP2B6^*∗*^4 (785A > G), and CYP2B6^*∗*^5 (1459C > T) were genotyped using tetra-primer amplification refractory mutation system polymerase chain reaction (ARMS–PCR). The amplification was conducted using 60 ng of extracted genomic DNA, 0.4 U Taq DNA polymerase (CinnaGen), six pmol of each primer, 10X PCR buffer, 0.5 mM dNTP, and 1.5 mM MgCl2. The mixture was initially denatured at 95°C for 3 minutes, followed by 32 cycles of 95°C for 1 minute, 56°C for 1 min, and 72°C for 2 min for CYP2B6 516G > T; 32 cycles of 95°C for 1 minute, 64°C for 50 seconds, and 72°C for 1 minute for CYP2B6 785A > G; 35 cycles of 95°C for 1 minute, 63°C for 1 min, and 72°C for 1 min for CYP2B6 1459C > T; followed by a final extension at 72°C for 10 min. Afterward, amplified fragments were run on a 1.5% agarose gel electrophoresis for 1 hour at 80 volt, ahead of staining using silver nitrate (CinnaGen) [19, 20].  Table 1 provides information on the primer sequences [6]. The DNA bands in the agarose gel were visualized under ultraviolet (UV) rays, and images were captured [19, 20].  Figure 2 schematically summarizes the genotyping process using Tetra-ARMS-PCR for CYP2B6^*∗*^6 (516G > T).

### 2.4. Statistical Analysis

Genotype and allele frequencies were carried out according to tetra-primer ARMS–PCR findings. The frequency of alleles and genotypes was accompanied by confidence intervals (CI) proportions, calculated based on the formula $\left(95\%\text{ CI}=p\pm \left(1.96\right.\times \sqrt{p\left(1-p\right)/n}\right)$. Chi-square analysis was used for comparing allele frequencies in different ethnicities [21, 22]. A *p* value less than 0.05 was considered statistically significant.

## 3. Results

### 3.1. Allelic and Genotype Frequency of CYP2B6^*∗*^6 (516G > T)

**Figure 3:**
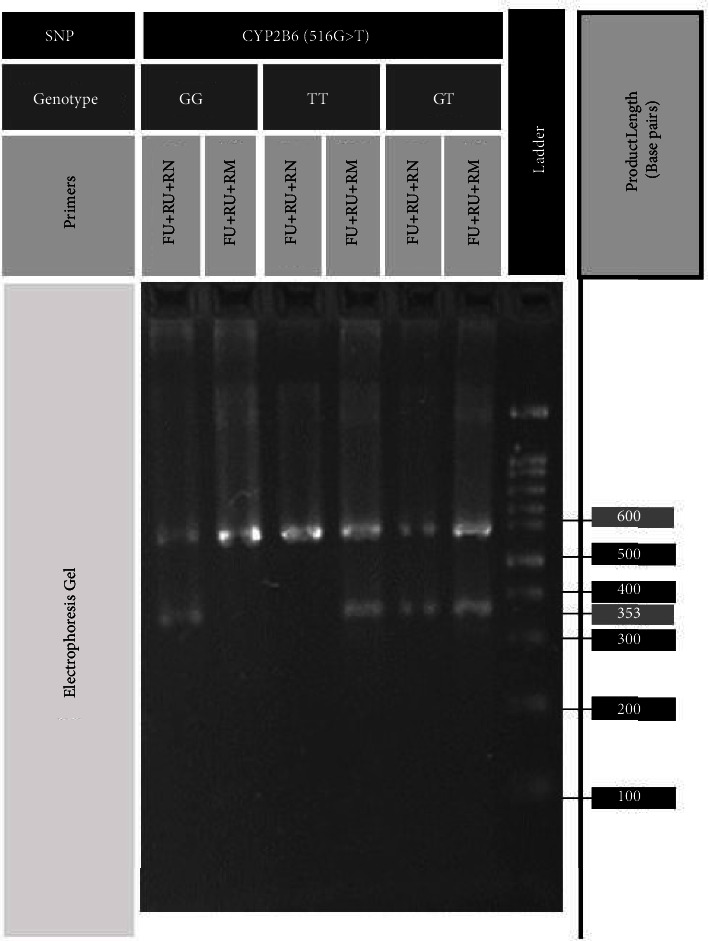
CYP2B6^*∗*^6 gene electrophoresis findings for three samples with genotypes of GG, TT, and GT from left to right. FU, forward outer primer; RU, reverse outer primer; RN, reverse nonmutant primer, RM, reverse mutant primer; the leftmost well is filled with a 100 bp DNA ladder.

Table 2 demonstrates the frequencies of CYP2B6^*∗*^6 (516G > T) genotypes in the Iranian population. The frequency of wild-type homozygotes is estimated to be 35.37% (95% CI: 27.67, 43.68), while the frequency of heterozygotes and mutated homozygotes was 46.26% (95% CI: 38.01, 54.66) and 18.37% (95% CI: 12.47, 25.59), respectively. The highest frequency of TT genotype was measured in the Kurd population (34.62%), whereas the lowest frequency was calculated in Turk participants (5.38%) (*p* value <0.05). It should be emphasized that Kurds' mutated allele frequency was 59.62% (95% CI: 45.10, 72.9), which was significantly higher than whole Iranian participants (*p* value <0.05). Allele frequencies of CYP2B6^*∗*^6 are available in Table 3. Also, an electrophoresis gel demonstrating different polymorphisms of CYP2B6^*∗*^6 is illustrated in Figure 3.

### 3.2. Allelic and Genotype Frequency of CYP2B6^*∗*^4 (785A > G)

The allelic and genotype frequency of CYP2B6^*∗*^4 in three major Iranian ethnicities is illustrated in Table 2. A great proportion of Iranians had wild-type homozygote genotypes for CYP2B6^*∗*^4 (43.54%, 95% CI: 35.39, 51.95). Furthermore, as shown in Table 3, mutated allele frequency was found to be 37.41% (95% CI: 31.87, 43.22) in the Iranian population. The highest and lowest G allele frequency was measured in Kurd (46.15, 95% CI: 32.23, 60.53) and Fars (28.98, 95% CI: 21.58, 37.31) participants (*p* value <0.05).

### 3.3. Allelic and Genotype Frequency of CYP2B6^*∗*^5 (1459C > T)

As Table 2 depicts data regarding CYP2B6^*∗*^5 (1459C > T) polymorphism, CC, CT, and TT frequency were 62.59%, 25.85%, and 11.56%, respectively. Among ethnicities, Turks showed the highest frequency for CC (76.92%, 95% CI: 63.16, 87.47), followed by 63.77% (95% CI: 51.31, 75.01) for Fars participants. On the other hand, Kurd participants showed a significantly higher frequency for the *T* allele (46.15%, 95% CI: 32.23, 60.53) compared to Iranians (62.59%, 95% CI: 54.23, 70.42) (*p* value <0.05). Frequencies of alleles in each ethnicity are summarized in Table 2.

## 4. Discussion

This study demonstrates the allele frequency of CYP2B6 mutations, which are clinically significant in the metabolism of efavirenz in three major Iranian ethnicities. The conclusion derived from the data obtained in the present study showed that Kurds, which comprise about 10 percent of the country's population, are found to be attributed at higher risk of both decreased and increased efavirenz metabolism compared to other Iranian ethnicities due to their higher frequency of CYP2B6^*∗*^6 and CYP2B6^*∗*^5, respectively.

As demonstrated in Figure 1, efavirenz pharmacokinetic is closely linked to CYP2B6 and its polymorphisms play a crucial role in the metabolization pace of the drug [13]. That is to say, the large intersubject variability of efavirenz exposure could be explained by the CYP2B6 genetic variations [23]. Clinical Pharmacogenetics Implementation Consortium (CPIC) guideline suggests CYP2B6 genotyping prior to prescription of efavirenz-containing antiretroviral therapy, as CYP2B6 is highly polymorphic and some of its variants lead to substantial differences in plasma efavirenz exposure [24]. For instance, CYP2B6^*∗*^6 (516G > T) results in aberrant splicing, which in turn leads to reduced CYP2B6 expression.  Table 4 summarizes the effects of CYP2B6^*∗*^6 (516G > T), CYP2B6^*∗*^4 (785A > G), and CYP2B6^*∗*^5 (1459C > T) polymorphisms in efavirenz metabolization and provides the frequencies of CYP2B6 polymorphisms.

Not only *in-vitro* studies demonstrated that CYP2B6^*∗*^6 (516G > T) is associated with the decreased catalytic activity of CYP2B6 and decreased efavirenz metabolism [35–37] but also clinical studies proved that CYP2B6^*∗*^6 (516G > T) is associated with increased efavirenz level in plasma [32, 38, 39]. In other words, previous studies revealed that populations with a higher frequency of CYP2B6^*∗*^6 are at higher risk of efavirenz toxicity. In this study, Kurds were identified as a population with CYP2B6^*∗*^6 mutated homozygote genotype (TT) frequencies as high as 34 percent.

The greatness of this percentage becomes more tangible when compared to previous studies. For instance, Haas et al.'s study reported a TT frequency of 20 percent in its African-Americans participants. They concluded that African-Americans have greater efavirenz plasma exposure during HIV therapy and should be considered a high-risk group in terms of efavirenz ADR [40]. The findings of the Gounden et al. study in South African HIV-infected patients were also similar to Haas et al.'s study [41]. On the other hand, studies conducted in Mozambican, Zimbabwean, and Senegalese populations reported a CYP2B6^*∗*^6 allele frequency higher than Kurd participants in this study [42–44]. To consider the study's small sample, we reported all frequencies with their 95% confidence intervals. It is noteworthy that even the lowest 95% confidence interval of TT frequency in the Kurd population is higher than the TT frequency reported in some previous studies [40, 45].

Zakeri et al.'s study, which is the single study conducted in Iran to evaluate the frequency of CYP2B6 variants, showed that CYP2B6^*∗*^6 allele frequency is 10.2% in the Baloch population in southeast Iran [44]. Their findings were consistent with our results in Turk and Fars populations. Conversely, the *T* allele was more frequent in the Kurd population than in the Baloch population. We should keep in mind that the Baloch ethnicity, which comprises only about 2 percent of the country's population [18], is not a representative sample of whole Iranians. Moreover, there is a significant geographical distance between Balochs predominantly living in the southeast and Kurds residence in the country's northwest.

Moreover, Zakeri et al. showed that the prevalence of mutated homozygote of CYP2B6^*∗*^4 (785A > G) (GG), which is attributed to decreased CYP2B6 activity, in Baloch ethnicity is 10.4 (7.8–13.8) percent [25], which is relatively lower than the results of this study. These findings can also be justified by the small share of Balochs in Iran's population and geographic considerations. On the other hand, the frequency of the G allele reported in this study is similar to other ethnic populations such as Timorians (29.2 percent) [46], Malays (37.2 percent) [47], and Indians (36.3 percent) [48].

Similar to CYP2B6^*∗*^6, the mutated allele frequency of CYP2B6^*∗*^5 (1459C > T) is higher in Kurds than in other Iranian ethnicities. TT phenotype leads to the increased catalytic activity of CYP2B6 and increased efavirenz metabolism [35]. Having a high frequency of both CYP2B6^*∗*^6 and ^*∗*^5 puts Kurds at higher risk of inadequate drug exposure and ADRs, prioritizing this ethnicity for testing CYP2B6 variants over other Iranian ethnicities. Arenaz et al. investigated the potential differences in allele frequencies of the CYP2B6 gene between Spaniards and Central Americans. They showed that the frequency of the *T* allele ranges from 1.0 percent in Japanese ethnicity to 14.0 in Caucasian (German) ethnicity. Although the frequency of CYP2B6^*∗*^5 in their study is not as high as ours, their findings verified that CYP2B6^*∗*^5 frequency is vastly different among ethnicities [49].

There are several limitations to our study. First, the sample size of the current study was small, and other cross-sectional studies with larger sample sizes should be conducted to evaluate the allele frequency in Iranian ethnicities and identify high-risk ethnicity groups. Moreover, in this study, three major mutations of CYP2B6 were evaluated (CYP2B6^*∗*^6 (516G > T), CYP2B6^*∗*^4 (785A > G), and CYP2B6^*∗*^5 (1459C > T)), while other minor mutations such as CYP2B6^*∗*^15 (1172T > A), CYP2B6^*∗*^11 (136A > G), CYP2B6^*∗*^2 (64C > T), and CYP2B6^*∗*^3 (777C > A) in CYP2B6 may also play a role in the drug metabolism. More importantly, sequence analysis was not conducted in this study. The lack of sequence analysis warns us to interpret the data of the investigated CYP2B6 polymorphisms more carefully.

In conclusion, this study revealed that a high frequency of CYP2B6^*∗*^6 and ^*∗*^5 leads to an increase in the risk of ADRs and insufficient anti-HIV response in Kurds, respectively. This study proposes genotyping for clinically significant mutations, especially in Kurds, before anti-HIV therapy with efavirenz.

## Figures and Tables

**Table 1:** Polymorphism details and primers.

rs	Allele	Nucleotide change	Amino acid change	Length of PCR products			Type		Primer sequences (5' > 3′)
rs3745274	CYP2B6^*∗*^6	(516G > T)	Gln172His	FU + RU = 600	FU + RN = 353	FU + RM = 353	Missense	FU	TGTGTTGCCTGGGTCTAAATC
								RU	CTGATTCTTCACATGTCTGCGT
								RN	AGCAGATGATGTTGGCGGTAATGGAC
								RM	TGATGTTGGCGGTAATGGAA

rs2279343	CYP2B6^*∗*^4	(785A > G)	Lys262Arg	FU + RU = 734	FU + RN = 256	FU + RM = 256	Missense	FU	GTTCCCATGGAGGGATTGGG
								RU	CTCTACACATCCAACCGCGTA
								RN	GAGCAGGTAGGTGTCGATGAGGTCCT
								RM	GTAGGTGTCGATGAGGTCCC

rs3211371	CYP2B6^*∗*^5	(1459C > T)	Arg487Cys	FU + RU = 381	FU + RN = 206	RU + FM = 209	Missense	FU	CACACTGGTGACCTTCTGTGT
								RU	CCTGCACTCACTTGCAATGT
								RN	CGCTTCCTGCCCCGCTGAAGGGGCTG
								FM	CAAAATACCCCCAACATACCAGATCT

**Table 2:** Genotype frequencies for CYP2B6 polymorphism in the Kurd, Turk, and Fars population.

CYP2B6^*∗*^6 (516G > T)				
Ethnicity		GG	GT	TT
Kurd (*n* = 26)	*n*	4	13	9
	Percent (95% CI)	15.38 (4.36, 34.87)	50.00 (29.93, 70.07)	34.62 (17.21, 55.67)
Turk (*n* = 52)	*n*	20	24	8
	Percent (95% CI)	38.46 (25.30, 52.98)	46.15 (32.23, 60.53)	15.38 (5.58, 25.19)
Fars (*n* = 69)	*n*	26	31	12
	Percent (95% CI)	37.68 (26.29, 50.17)	44.93 (32.92, 57.38)	17.39 (9.32,28.41)
Total (*n* = 147)	*n*	52	68	27
	Percent (95% CI)	35.37 (27.67, 43.68)	46.26 (38.01, 54.66)	18.37 (12.47, 25.59)

CYP2B6^*∗*^4 (785A > G)				
Ethnicity		AA	AG	GG
Kurd (*n* = 26)	*n*	10	8	8
	Percent (95% CI)	38.46(20.23, 59.43)	30.77 (14.33, 51.79)	30.77 (14.33, 51.79)
Turk (*n* = 52)	*n*	20	18	14
	Percent (95% CI)	38.46 (25.30, 52.98)	34.61 (21.97, 49.09)	26.92 (15.57, 41.02)
Fars (*n* = 69)	*n*	34	30	5
	Percent (95% CI)	49.28 (37.02, 61.59)	43.48 (31.58, 55.96)	7.25 (2.39, 16.11)
Total (*n* = 147)	*n*	64	56	27
	Percent (95% CI)	43.54 (35.39, 51.95)	38.10 (30.22, 46.46)	18.37 (12.47, 25.59)

CYP2B6^*∗*^5 (1459C > T)				
Ethnicity		CC	CT	TT
Kurd (*n* = 26)	*n*	8	12	6
	Percent (95% CI)	30.77 (14.33, 51.79)	46.15 (26.59, 66.63)	23.07 (8.97, 43.65)
Turk (*n* = 52)	*n*	40	8	4
	Percent (95% CI)	76.92 (63.16, 87.47)	15.38 (6.88, 28.08)	7.69 (2.14, 18.54)
Fars (*n* = 69)	*n*	44	18	7
	Percent (95% CI)	63.77 (51.31, 75.01)	26.08 (16.25, 38.06)	10.14 (4.18, 19.79)
Total (*n* = 147)	*n*	92	38	17
	Percent (95%CI)	62.59 (54.23, 70.42)	25.85 (18.99, 33.71)	11.56 (6.88, 17.87)

**Table 3:** Allele frequencies in Iranian populations.

CYP2B6^*∗*^6 (516G > T)				
Ethnicity	G	T	*χ*2 statistic	*p* value
Total (*n* = 147)	58.50 (52.64, 64.19)	41.50 (35.81, 47.36)		
Kurd (*n* = 26)	40.38 (27.01, 54.90)	59.62 (45.10, 72.99)	5.8808	0.015^*∗*^
Turk (*n* = 52)	61.54 (51.49, 70.91)	38.46 (29.09, 48.51)	0.2932	0.588
Fars (*n* = 69)	60.14 (51.47, 68.38)	39.86 (31.62, 48.53)	0.1046	0.746

CYP2B6^*∗*^4 (785A > G)				
Ethnicity	A	G	*χ*2 statistic	*p* value
Total (*n* = 147)	62.59 (56.78, 68.13)	37.41 (31.87, 43.22)		
Kurd (*n* = 26)	53.85 (39.47, 67.77)	46.15 (32.23, 60.53)	1.422	0.233
Turk (*n* = 52)	55.77 (45.70, 65.50)	44.23 (34.50, 54.30)	1.4975	0.221
Fars (*n* = 69)	71.01 (62.69, 78.42)	28.98 (21.58, 37.31)	2.9442	0.086

CYP2B6^*∗*^5 (1459C > T)				
Ethnicity	C	T	*χ*2 statistic	*p* value
Total (*n* = 147)	75.51 (70.18, 80.32)	24.49 (19.68, 29.82)		
Kurd (*n* = 26)	53.85 (39.47, 67.77)	46.15 (32.23, 60.53)	10.3442	0.0012^*∗*^
Turk (*n* = 52)	84.62 (76.22, 90.94)	15.38 (9.06, 23.78)	3.6983	0.054
Fars (*n* = 69)	76.81 (68.87,83.57)	23.19 (16.43, 31.13)	0.087	0.768

^*∗*^ *p* value for *χ*2 test is statistically significant (*p* value <0.05).

**Table 4:** The effects of CYP2B6^*∗*^6 (516G > T), CYP2B6^*∗*^4 (785A > G), and CYP2B6^*∗*^5 (1459C > T) polymorphisms in efavirenz metabolization and mutated allele frequency of CYP2B6 polymorphisms worldwide, especially in West Asia.

Polymorphism	CYP2B6	Efavirenz metabolization	Proposed mechanism	Studies' findings (mutated allele percentage)							
				Current study	Iran (Mazani) [6]	Iran (Baloch) [25]	West Asian [26]	Italian [27]	Caucasian [28]	German [29]	United Kingdom [30]
CYP2B6^*∗*^6 (516G > T)	Reduced expression [31, 32]	Poor metabolization	(i) Aberrant splicing [33] (ii)Higher protein expression in COS-1 cells [34] (iii) Demethylation catalytic activity with the substrate 4-trifluoromethylcoumarin [34]	41.50	48	10.4	21.5	29.1	25.6	25	28.1

CYP2B6^*∗*^4 (785A > G)	Reduced expression [32]	Poor metabolization	(i) Higher protein expression in COS-1 cells [34] (ii) Demethylation catalytic activity with the substrate 4-trifluoromethylcoumarin [34]	37.41	43	23.1	14.7	2.82	4	5	1.1

CYP2B6^*∗*^5 (1459C > T)	Increased activity [35]	Rapid metabolization	(i) Unknown.	24.49	0.08	2.4	NR	17.3	10.9	9.5	12.2

COS, CV-1 (simian) in origin and carrying the SV40 genetic material; NR, not reported.

## Data Availability

The raw data supporting the conclusions of this article will be made available by the corresponding authors without undue reservation.
